# Pulmonary Infections Caused by Emerging Pathogenic Species of *Nocardia*

**DOI:** 10.1155/2019/5184386

**Published:** 2019-10-01

**Authors:** Harish Manoharan, Sribal Selvarajan, K. S. Sridharan, Uma Sekar

**Affiliations:** Sri Ramachandra Institute of Higher Education and Research, Chennai, India

## Abstract

Pulmonary infections are the most common clinical manifestations of *Nocardia* species. There is an increase in cases of nocardial infections occurring worldwide attributable to the increase in the immunosuppressed population. The availability of molecular methods has aided the detection of more number of cases as well as unusual species. Still, it remains one of the most underdiagnosed pathogens. Recognition of drug resistance in this organism has now mandated early and precise identification with speciation for effective treatment and management. Nocardial species identity can predict antimicrobial susceptibility and guide clinical management. Here, we report two cases of pulmonary nocardiosis caused by unusual species of *Nocardia*, namely, *N. cyriacigeorgica* and *N. beijingensis* identified by 16S rRNA gene-based sequencing. These cases are being reported for their rarity.

## 1. Background

Nocardiosis is usually an opportunistic infection, and systemic immunosuppression, particularly cell-mediated immunity dysfunction, predisposes patients to infection. The most common presentation is pulmonary infection; dissemination to other organ systems is a common complication in progressive disease. Mortality appears to correlate with the causative species and the site of infection and can be as high as 50% in patients with disseminated diseases [1].

Nocardiae are aerobic actinomycetes ubiquitously found in soil and aquatic habitats. They are thin, aerobic, Gram-positive bacilli that form branching filaments. The bacteria stain irregularly and appear beaded on Gram stain [2]. The original species identification was based on the ability to use specific nutrients and to decompose substrates such as adenine, casein, urea, gelatin, and xanthine. However, gene sequencing and deoxyribonucleic acid DNA-DNA hybridization have now defined the true taxonomy. *Nocardia asteroides* was previously reported to be the most common cause of human disease [3]. The number of species causing human disease is large, and the most frequently reported species include *N. abscessus*, *N. brevicatena/paucivorans* complex, *N. nova* complex, *N. transvalensis* complex, *N. farcinica*, *N. otitidiscaviarum*, *N veterana*, *N. brasiliensis*, and *N. pseudobrasiliensis*.

The common predisposing factors for nocardial infection are long-term steroid use, neoplastic disease, and human immunodeficiency virus (HIV) infection. The pathogenic virulence of *Nocardia* species is low, and therefore fewer cases are reported among the immunocompetent patients. Major setback in the diagnosis of *Nocardia* is that they do not have any specific clinical manifestations and no pathognomonic features either radiologically or histopathologically [4].

Growth in culture may be delayed and missed especially when other commensals are present since they overgrow and retard nocardial growth. There are no specific guidelines for the antimicrobial susceptibility testing, and hence it is advisable to select antibiotics on the basis of molecular taxonomy.

Conventional culture and species identification in the bacteriology laboratory is very time consuming, and the accurate species identification is often not possible. Routine laboratory algorithms for the phenotypic identification of *Nocardia* species are limited in practice. Usage of molecular identification methods like 16S rRNA gene sequencing plays a very vital role in rapid detection of the species and will help in better management of nocardiosis. hsp65 sequence analysis also is an alternative molecular tool for species identification [3]. Although the former is widely used for identifying this group of organisms, hsp65 has more microheterogeneity regions compared to 16S rRNA and can be better discriminatory, but only few sequences of hsp65 gene of *Nocardia* species are available in public databases for comparisons which limits its use in identification. So, currently 16S rRNA gene sequencing is considered as a better method for identification of *Nocardia* to the species level [5–7]. Few studies have been conducted in the past to compare hsp65 and 16S rRNA regions of *Nocardia* with respect to the standard strains. On comparison of these two gene sequences, the mean percentage dissimilarity in identification was found to be higher with the hsp65 gene sequences. Hence in this study, 16S rRNA was used for species detection.

DNA-DNA hybridization was once considered as the gold standard for diagnosis of *Nocardia* species, though not widely used now. Other molecular techniques used are sequence analysis of RNA polymerase (rpoB), gyrase B of the *β*-subunit of DNA topoisomerase (gyrB), and secA preprotien translocase-subunit A (secA1) regions. PCR-RFLP and MALDI-TOF MS are also being advocated in the recent era [7].

## 2. Case 1

**Figure 1:**
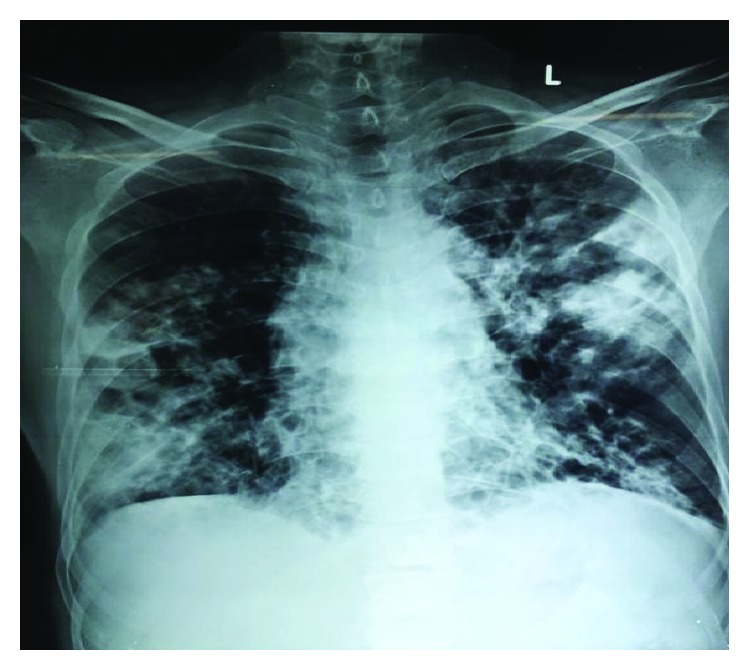
Chest x-ray showing bilateral midzone and lower zone consolidation.

A 40-year-old male presented to the outpatient clinic of the tertiary care hospital with complaints of cough, expectoration, hemoptysis, and fever off and on particularly in the evenings. He had been treated for pulmonary tuberculosis previously in the year 2009 and subsequently in the year 2012 following its remission. He was on oral and inhalational steroids for several years for wheeze-like symptoms. He had sought consultation and had been admitted in other hospitals several times for similar complaints. The patient did not have any other comorbid conditions. He was a welder by occupation and so exposure to fumes and fine metallic dust particles was noted as a significant factor in the clinical history. Physical examination of the respiratory system revealed bilateral coarse crepitations. Examination of other systems did not reveal any contributory findings. Chest radiograph and routine blood workup were undertaken. Chest X-ray revealed bilateral midzone and lower zone consolidation (Figure 1). With a diagnosis of bilateral bronchiectasis, he was admitted to the hospital for further evaluation and to investigate the status of pulmonary tuberculosis in the light of hemoptysis.

**Figure 2:**
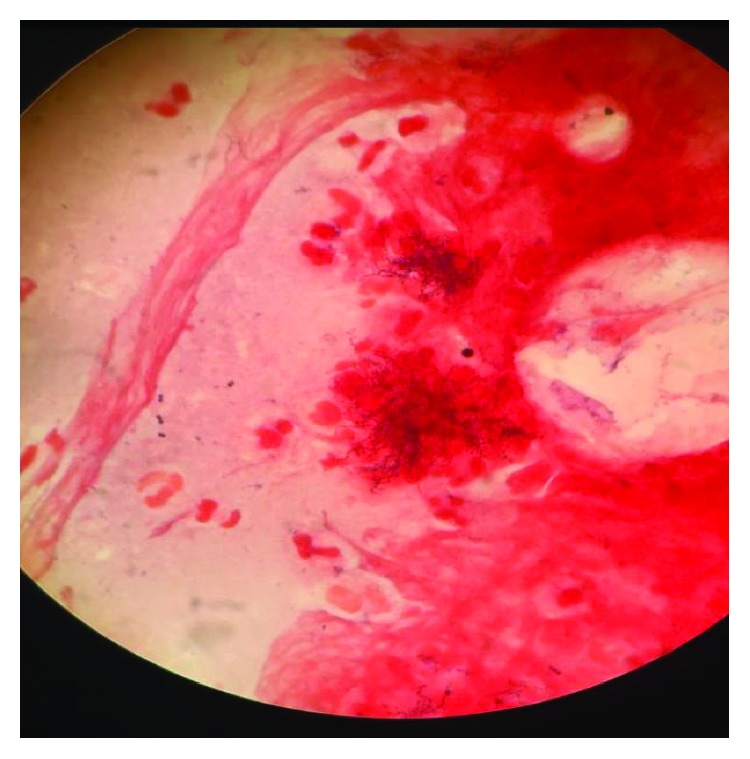
Gram stain showing branching Gram-positive bacilli suggestive of *Nocardia*.

**Figure 3:**
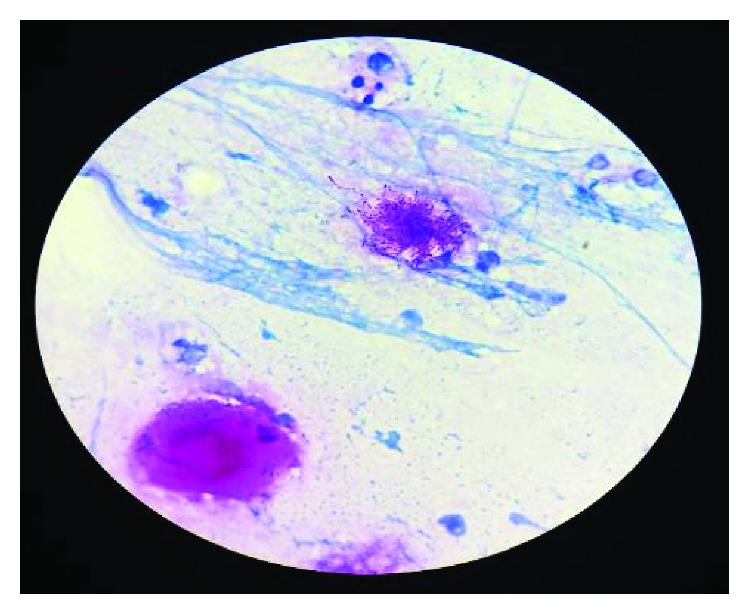
1% acid-fast staining showing weakly acid-fast slender and filamentous bacilli conforming *Nocardia*.

**Figure 4:**
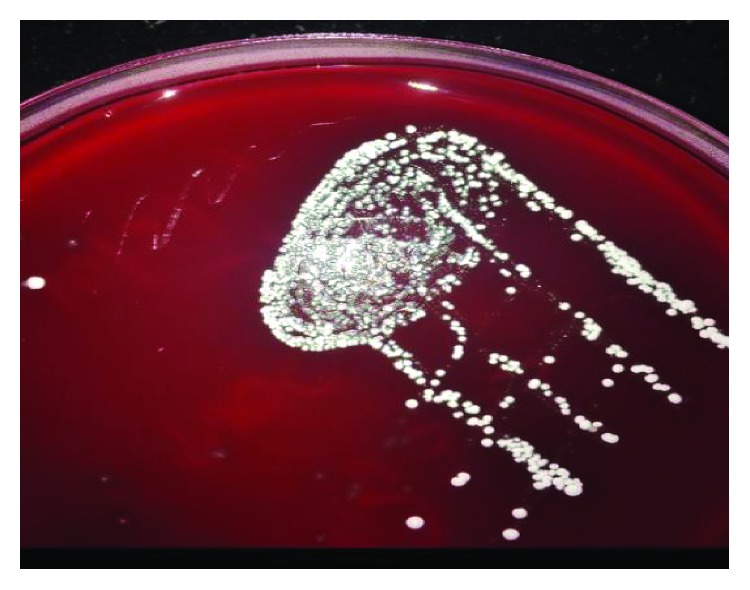
5% sheep blood agar plate showing dry chalky white colonies of *Nocardia*.

The patient was initially started on intravenous piperacillin/tazobactam for empiric treatment of community-acquired secondary pulmonary infection. Despite the antibiotic, the patient had sustained decrease in oxygen saturation leading to deterioration in pulmonary function over the next few days. With impending respiratory failure, he was shifted to the Intensive Care Unit (ICU). The antibiotic was escalated to meropenem due to his deteriorating clinical condition. Blood and urine cultures were sterile, and 20% acid-fast staining of sputum and respiratory secretion was also negative. Sputum was sent for bacterial culture. The culture plates initially had scanty growth of normal flora, but on Gram stain there were few branching Gram-positive bacilli observed which was suggestive of *Nocardia* (Figure 2). In view of this, modified acid-fast staining with 1% acid was performed on the smear, and it revealed plenty of weakly acid-fast branching slender and filamentous bacilli characteristic of *Nocardia* (Figure 3). The culture media on further incubation of 72 hours yielded dry chalky white colonies (Figure 4). Gram's stain and acid-fast stain of these colonies confirmed them as *Nocardia*. For species identification, 16S rRNA gene sequencing was undertaken. BLAST search of the sequence was done using the taxonomy browser of the National Center for Biotechnology Information (NCBI). The 662 bp of the sequence revealed a 100% match with *Nocardia cyriacigeorgica*. The sequence has been submitted to GenBank with accession number MK641487.

*Nocardia cyriacigeorgica* belongs to *Nocardia asteroides* complex (vi). This species was first described in 2001, and strains of *N. cyriacigeorgica* have since been recovered as the etiologic agent of human infection in Western Europe, Greece, Turkey, Japan, Thailand, and Canada [1]. Most cases of infection have occurred in the context of HIV-related or iatrogenic immune suppression. Pulmonary nocardiosis caused by *Nocardia cyriacigeorgica* in patients with *Mycobacterium avium* complex lung disease has been described before [8]. It has also been identified as the causative agent of an anterior mediastinal abscess in a patient with preexisting lung disease [9] and the aetiological agent of native valve endocarditis in a patient with chronic obstructive pulmonary disease (COPD) [10].

In addition to sulfonamide susceptibility, they are generally susceptible to broad-spectrum cephalosporins, amikacin, imipenem, and linezolid but resistant to penicillin, clarithromycin, and ciprofloxacin. It has been reported in the literature that serious life-threatening infections caused by *Nocardia cyriacigeorgica* are controlled well with dual therapy [1]. In view of the species identification, the patient was started on injection imipenem and oral trimethoprim sulphamethoxazole. The patient began to improve clinically with this therapy. Oxygen saturation levels improved, fever declined, and the patient was shifted out of the Intensive Care Unit. Subsequently, with sustained improvement, he was discharged from the hospital in good health with the advice to continue oral cotrimoxazole for six months. The patient continues to remain relapse free.

## 3. Case 2

**Figure 5:**
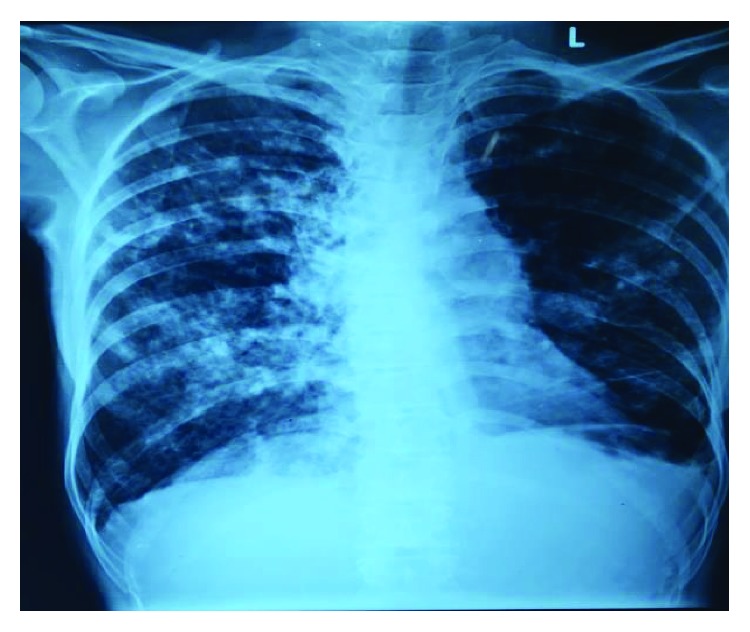
Chest x-ray showing bilateral midzone and lower zone consolidation more on the right side along with hyperlucency in the left upper zone due to left lower lobe lobectomy.

A 28-year-old female belonging to lower socioeconomic class, presented at the outpatient clinic with complaints of shortness of breath and cough with expectoration accompanied by episodes of fever. She had been diagnosed earlier with bilateral bronchiectasis and had left lower lobectomy of the lung performed four years prior to this presentation. She had been hospitalized several times for similar complaints. On detailed elicitation of clinical history, the patient informed that she has had symptoms pertaining to the respiratory tract since the age of two and had been investigated several times for pulmonary tuberculosis but with negative results each time. On examination, she looked ill built and emaciated. Chest auscultation revealed bilateral crepitations. Chest x-ray shows bilateral mid and lower zone consolidation, more on the right side along with compensatory hyperlucency in the left upper zone due to left lower lobe lobectomy (Figure 5). She was hospitalized for management of presumptive pulmonary infection and to evaluate the other causes of fever if any. Patient was started on empirical treatment with intravenous piperacillin/tazobactam 4.5 gm IV TDS. Routine blood workup did not reveal any abnormality, and blood and urine cultures were sterile. Sputum was sent for culture and smear examination with modified acid-fast staining. The smear revealed weakly acid-fast branching filamentous bacilli characteristic of *Nocardia*. Gram's stain also showed the presence of Gram-positive branching bacilli. The organism grew in culture after 72 hours of incubation. For species identification, 16S rRNA gene sequencing was done, and a BLAST search of the sequence was done using the taxonomy browser of the National Center for Biotechnology Information (NCBI). The 732 bp of the sequence revealed a 99.32% match with *Nocardia beijingensis* species. The sequence was submitted to GenBank with accession number MK641488. The patient was started on oral cotrimoxazole monotherapy. She improved considerably, and the fever subsided. Dyspnoea improved dramatically. She was discharged after three weeks of therapy with advice to continue oral cotrimoxazole.

*N. beijingensis* infections have been described in both immunocompetent and immunosuppressed hosts [11]. Disseminated infection has been described in immunocompetent hosts [12]. Pneumonia, pulmonary abscess, and endobronchial lesions are the other kind of clinical presentations associated with this species. *N. beijingensis* was first described by Wang et al. from a soil sample in a sewage ditch at Xishan Mountain in Beijing [13]. Strains have been isolated in Asia between 2004 and 2010 from patients with pulmonary nocardiosis and in immunocompromised patients.

## 4. Conclusion

Molecular techniques offer a distinct advantage for identification of *Nocardia* species. There is a need for heightened awareness to identify *Nocardia* in clinical samples for early diagnosis and treatment. Since pulmonary nocardiosis mimics tuberculosis, its identification is crucial in countries where tuberculosis is endemic. Therefore, indiscriminate usage of antituberculosis drugs can be curtailed. To conclude, improved recognition and effective implementation of anti-infective therapy for pulmonary nocardiosis will reduce mortality and improve the outcomes in patient management.

## 5. Limitations

This study report is based on two patients only. Further studies are required with more number of patients for better understanding of the outcomes and prognosis of the illness.
